# Curcumin-mediated bone marrow mesenchymal stem cell sheets create a favorable immune microenvironment for adult full-thickness cutaneous wound healing

**DOI:** 10.1186/s13287-018-0768-6

**Published:** 2018-01-31

**Authors:** Zhi Yang, Chengmin He, Jinyang He, Jing Chu, Hanping Liu, Xiaoyuan Deng

**Affiliations:** 1 MOE Key Laboratory of Laser Life Science, College of Biophotonics, South China Normal University, No. 55 Zhongshan Avenue West, Tianhe District, Guangzhou, 510631 China; 2 Tropical Medicine Institute, Guangzhou University of Chinese Medicine, Guangzhou, China

**Keywords:** Curcumin, Bone marrow mesenchymal stem cell, Cell sheet, Immune response, Cutaneous wound healing

## Abstract

**Background:**

Adult full-thickness cutaneous wound repair suffers from an imbalanced immune response, leading to nonfunctional reconstructed tissue and fibrosis. Although various treatments have been reported, the immune-mediated tissue regeneration driven by biomaterial offers an attractive regenerative strategy for damaged tissue repair.

**Methods:**

In this research, we investigated a specific bone marrow-derived mesenchymal stem cell (BMSC) sheet that was induced by the Traditional Chinese Medicine curcumin (CS-C) and its immunomodulatory effects on wound repair. Comparisons were made with the BMSC sheet induced without curcumin (CS-N) and control (saline).

**Results:**

In vitro cultured BMSC sheets (CS-C) showed that curcumin promoted the proliferation of BMSCs and modified the features of produced extracellular matrix (ECM) secreted by BMSCs, especially the contents of ECM structural proteins such as fibronectin (FN) and collagen I and III, as well as the ratio of collagen III/I. Two-photon fluorescence (TPF) and second-harmonic generation (SHG) imaging of mouse implantation revealed superior engraftment of BMSCs, maintained for 35 days in the CS-C group. Most importantly, CS-C created a favorable immune microenvironment. The chemokine stromal cell-derived factor 1 (SDF1) was abundantly produced by CS-C, thus facilitating a mass migration of leukocytes from which significantly increased expression of signature T_H_1 cells (interferon gamma) and M1 macrophages (tumor necrosis factor alpha) genes were confirmed at 7 days post-operation. The number of T_H_1 cells and associated pro-inflammatory M1 macrophages subsequently decreased sharply after 14 days post-operation, suggesting a rapid type I immune regression. Furthermore, the CS-C group showed an increased trend towards M2 macrophage polarization in the early phase. CS-C led to an epidermal thickness and collagen deposition that was closer to that of normal skin.

**Conclusions:**

Curcumin has a good regulatory effect on BMSCs and this promising CS-C biomaterial creates a pro-regenerative immune microenvironment for cutaneous wound healing.

## Background

Adult full-thickness cutaneous wounds, caused by burning, mechanical injury, or chronic ulcers, and so forth, usually lead to a non-functional tissue known as a scar [1, 2]. Wound healing is a complex biological process in which the immune response plays a vital role in host defense, necrotic cell clearance, and tissue regeneration. Indeed, a dysregulated immune response can lead to the development of pathological fibrosis or scarring, even impairing normal tissue function and ultimately leading to restoration failure [3]. The most conspicuous and often-reported feature of chronic inflammatory is the persistent presence of elevated levels of pro-inflammatory cytokines, such as tumor necrosis factor (TNF)-α, interleukin (IL)-1β, and IL-6, secreted by type I immune cells (T_H_1 cells) and associated pro-inflammatory M1 macrophages [4]. However, the depletion of macrophages in early immune injury leads to failures in matrix degradation during tissue repair [5]. In contrast, the last decade has seen the opposite side of the immune response, showing that a suitable immune response can be an effective strategy for promoting tissue regeneration [6]. Tissue-repairing M2 macrophages possess anti-inflammatory properties and support the functions of T helper 2 (T_H_2)-associated effector cells [7]. These two types of cells work together to promote tissue repair by expressing several wound healing factors such as arginase, platelet-derived growth factor (PDGF), vascular endothelial growth factor (VEGF)α, and insulin-like growth factor (IGF) [8–10].

Immune mediation driven by biomaterial is regarded as an innovative tissue regenerative strategy. There have been numerous attempts to seek promising biomaterials to improve cutaneous wound healing. Bone marrow-derived mesenchymal stem cells (BMSCs), based on their key roles during the wound healing process, are considered an attractive therapeutic biomaterial [11, 12] and exhibit excellent potential for reconstituting tissue due to their self-renewal ability, differentiation into various cell lineages, and secretion of paracrine factors [13]. More importantly, investigations in the past few years have provided new insight into the function of BMSCs during immune response in tissue repair. The immunoregulatory properties of BMSCs are appealing for their ability to inhibit T-cell differentiation into T_H_1 cells and the switch of macrophages from a pro-inflammatory type 1 (M1) to an anti-inflammatory type 2 (M2) phenotype [14–16]. However, therapies based on stem cells alone limit their functions and effectiveness in the clinic. The administration of BMSCs by intravenous injection or topical delivery results in a low delivery number or survival rate in the wound site due to enzymatic digestion of the single cell suspension in vitro and the lack of an appropriate carrier to protect BMSCs in the wound area [17].

BMSC sheet technology has emerged as an attractive approach that has been applied in many clinical treatments, such as wound healing, periodontal-like tissue regeneration, cardiac healing, and osseointegration [18–22]. Prolonged cultivation and protection against enzyme digestion allows increased cell numbers and long-term viability. Moreover, the BMSC sheet can provide an intact biomaterial consisting of cultured cells and secreted cytokines, along with natively deposited extracellular matrix (ECM). Through an intricate understanding of wound biology that has been developed by researchers over past decade, the importance of the ECM has been fully recognized. Far beyond acting only as a structural support, the ECM provides cells with a dynamic biophysical, biomechanical, and biochemical niche, and exerts tailored biological activities on cells to manipulate their behaviors [23].

Inspired by the immunoregulatory properties of BMSCs and the procedures of constituting an intact BMSC sheet (inducing BMSCs to secret a vast number of biological cytokines and produce native ECM), we envisage that by effectively mediating BMSCs to selectively secrete cytokines and produce specified endogenous ECM, a new biomaterial capable of regulating the immune response could be established and could promote wound healing. Some molecules have regulatory functions for directing the behavior of BMSCs. Traditional Chinese Medicine (TCM) can activate cells for wound regeneration through adjuvants derived from their ingredients [24]. Mounting evidence has shown that TCM as a novel therapeutic material has a powerful therapeutic effect on many types of wound healing, such as periodontal ligament defects and chronic ulcers, among others [25, 26]. Curcumin, the extract of zingiberaceae roots, has been widely used as a medicine for various diseases for many centuries. Curcumin exhibits a broad range of wound healing properties, such as anti-oxidant, anti-inflammatory, anti-infective, and anti-fibrosis effects [27–29]. Therefore, curcumin was initiated as a regulatory molecule to mediate BMSCs to produce a new, specific type of BMSC sheet. In this research, we focused on investigating the functional role of curcumin, particularly its ability to regulate BMSC behavior and ECM production, and hence to create a favorable immune microenvironment for adult full-thickness cutaneous wound healing.

## Methods

### Ethics statement

All experimental protocols were approved by the Ethical Committee for Animal Experiments of South China Normal University. All animal experiments conducted in this research were performed in accordance with the guidelines of South China Normal University Intramural Animal Use and Care Committee and met the NIH guidelines for the care and use of laboratory animals.

### Experimental animals

Four-week-old green fluorescent protein (GFP)^+^ C57BL/6 mice purchased from Cyagen Biosciences (Guangzhou, China) were used for culturing BMSCs and BMSC sheets. Ten-week-old male BALB/c mice were purchased from the Experimental Animal Center of Southern Medical University (Guangzhou, China).

To evaluate the effects of curcumin on the quality of wound repair, mice were randomly assigned to three different treatment groups. The control group received saline. The BMSC-curcumin sheet group (CS-C) received the BMSC sheet induced with curcumin applied to the affected area. The BMSC sheet group (CS-N) received the BMSC sheet induced without curcumin applied to the affected area. All mice were housed under pathogen-free conditions (22 °C, 12-h light/12-h dark cycles and 50–55% humidity) with proper food and water. All surgical procedures were performed under general anesthesia via an intraperitoneal injection of 1% pentobarbital.

### Construction of BMSC sheets and induction with curcumin

GFP^+^ BMSCs were obtained as previously reported by our group [30]. BMSC sheets were prepared by plating 1.5 × 10^5^ of third passage cells on culture dishes (six-well plates, Corning). BMSC sheets were cultured in OriCell™ mouse MSC Growth Medium (MUCMX-90011, Cyagen Biosciences, Inc.) with 100 mg/mL vitamin C in a humidified incubator (Thermo Scientific Forma 3110, Thermo Fisher Scientific, Inc.) at 37 °C with 5% CO_2_. The experimental group was additionally subjected to 0.5 μM curcumin throughout the entire incubation stage. The culture medium was changed every 2 days. The culture was then continued for 12 days until a white membrane structure could be observed and could be detached intact from the substratum using a cell scraper (Biologix, Angfei Biosciences, Inc.).

### Scanning electron microscopy (SEM) imaging of BMSC sheets

CS-N and CS-C were first fixed in 2.5% glutaraldehyde at 4 °C for 4 h. They were then cleaned four to six times in 0.1% phosphate buffer and dehydrated in a gradient of alcohol concentrations (30%, 50%, 70%, 90%, 100%). Finally, the samples were freeze-dried and sputter-coated with a 30-nm gold layer. SEM images are obtained using a scanning electron microscope (Zeiss Ultra 55, Carl Zeiss, Jena, Germany). The arrangement of cells and collagen was clearly visible.

### Wound model and surgical procedure

Ten-week-old male BALB/c mice were anesthetized with an intraperitoneal injection of pelltobarbitalum natricum. The hair on the dorsal skin of the mice was shaved and removed and the bared skin was sterilized with 75% alcohol. A full-thickness, 7-mm diameter skin wound was produced using a biopsy punch. A CS-C sheet, CS-N sheet, or saline (control) was then placed on the wound site (depending on the group assigned). Finally, the wound area was covered with Comfeel transparent dressing (Coloplast, Beijing, China) and wrapped with medical adhesive tape.

### Histological and immunofluorescence analysis

The wound bed tissues were harvested at 3, 7, 14, 21, and 28 days after surgery, and the two cell sheet group tissues were fixed with 4% paraformaldehyde and then embedded in paraffin and cut into 5-mm thick longitudinal sections. The sections were subjected to hematoxylin and eosin (H&E) staining for histological analysis of wound regeneration. In addition, Masson staining was carried out to reveal the collagen accumulation state. For immunofluorescence staining, to investigate the in vivo immune reaction of curcumin treatment in the different groups, anti-CD45, anti-CD11c, anti-IFN-γ, anti-Relmα, anti-SDF1, anti-TGFβ, and anti-IL1RN antibodies (Abcam) were used to identify lymphocytes, M1 macrophages, T_H_1 cells, M2 macrophages, stromal cell-derived factor 1 (SDF1)^+^ cells, transforming growth factor (TGF)β, and IL-1 receptor antagonist (IL1RN). To investigate the contents of the cell sheets, sections were incubated with antibodies against type I collagen (Col-I, 1:50, Abcam) and type III collagen (Col-III, 1:80, Abcam). Secondary antibodies were goat anti-rabbit IgG (Alex Fluor488 conjugated, 1:200, CST) and goat anti-rat IgG (Alex Fluor555 conjugated, 1:500, CST). All data were analyzed using ImageJ software.

### Real-time polymerase chain reaction (RT-PCR) analysis

Total RNA from newly formed tissue was isolated with TRIzol Reagent (TaKaRa, Dalian, China). The samples were then used for cDNA synthesis using the PrimeScript® RT reagent Kit with gDNA Eraser (TaKaRa, Dalian, China), and real-time PCR was carried out according to the manufacturer’s instructions (Applied Biosystems 7500) with SYBR® Premix Ex Taq II (TaKaRa, Dalian, China). The primer sequences for each gene are described in Table 1. All genes were assessed using the 2^– ∆∆Ct^ method.

**Table 1:** Primers used for real-time polymerase chain reaction

Gene	Primer sequence
*Col-1*	Forward 5’- AAGAAGACATCCCTGAAGTCA -3’
	Reverse 5’- GCAGATACAGATCAAGCATACC -3’
*Col-3*	Forward 5’- GCAAGGCAATGAGACTACC -3’
	Reverse 5’- CCAATGTCCACACCAAATTC -3’
*Il-4*	Forward 5’- CTAGTTGTCATCCTGCTCTTCT -3’
	Reverse 5’- CTTCTCCTGTGACCTCGTTC -3’
*Tnfα*	Forward 5’- AGGTTCTCTTCAAGGGACAA -3’
	Reverse 5’- CCTGGTATGAGATAGCAAATCG -3’
*Ifnγ*	Forward 5’- ATGAACGCTACACACTGC -3’
	Reverse 5’- CCACATCTATGCCACTTGAG -3’
*Relmα*	Forward 5’- TACTGGGTGTGCTTGTGGCTTTGC -3’
	Reverse 5’- GGCAGTGGTCCAGTCAACGAGTAAG -3’
*Actinβ*	Forward 5’- CGTTGACATCCGTAAAGACC -3’
	Reverse 5’- TAGGAGCCAGAGCAGTAATC -3’
*Cxcr4*	Forward 5’- CTCCTCCTGACTATACCTGAC-3’
	Reverse 5’- CGAGACCCACCATTATATGC -3’
*Tgfβ*	Forward 5’- CTGCTGACCCCCACTGATAC -3’
	Reverse 5’- AGCCCTGTATTCCGTCTCCT -3’
*IL1RN*	Forward 5’- ACCTTCATAGTGTGTTCTTGG -3’
	Reverse 5’- CTTCTTCTTTGTTCTTGCTCAG -3’
*Arg1*	Forward 5’- GAAGAATGGAAGAGTCAGTGTG -3’
	Reverse 5’- GGAGTGTTGATGTCAGTGTG -3’
*Jag2*	Forward 5’- GTGTGGTTATCTGCGTATGG -3’
	Reverse 5’- GTTGCGGATGGGATTGAG -3’
*iNOS*	Forward 5’- CCTATCTCCATTCTACTACTACCA G -3’
	Reverse 5’- ACCACTTTCACCAAGACTCTA -3’
*Tbx21*	Forward 5’- CGCATCTGTTGATACGAGTG -3’
	Reverse 5’- TGGTTGGATAGAAGAGGTGAG -3’

### DNA amplification and sequencing

To determine the GFP^+^ cell activity and quantity in vivo, skin samples were harvested at 0, 21, 28, and 35 days after surgery. Total DNA from the samples was isolated with the TIANamp Genomic DNA kit (DP130227, TIANGEN Biotech, Beijing, China). The DNA was then amplified using TaKaRa Ex Taq® (TaKaRa, Dalian, China), including 0.25 μL TaKaRa Ex Taq (5 U/μL), 4.0 μL dNTP mixture, 5 μL 10× Ex Taq buffer (Mg^2+^ Plus, 2.0 μL template DNA, 3.0 μL each of upstream and downstream primers, and 32.75 μL sterile distilled water). The GFP primer sequences were: forward 5’-GAAGAACGGCATCAAGGT-3’, reverse 5’-GCTCAGGTAGTGGTTGTC-3’. After obtaining the DNA amplification products, the products were sent to Meiji biological company (Shanghai, China) for sequencing.

### Intravital imaging of BMSC sheets transplanted into the wound site ex vivo

To evaluate the GFP^+^ cell activity and trace the cells of the GFP-BMSC-sheets in vivo, second-harmonic generation (SHG) imaging and two-photon fluorescence (TPF) imaging with a commercial LSM 710 NLO confocal microscope (Zeiss, Jane, Germany) was conducted. The whole process is quick so that the samples remain alive. The process captures a three-dimensional image using the stack scan mode.

### Flow cytometry

To explore the effects of curcumin on cell proliferation, the curcumin treatment group was analyzed at 1, 3, 6, and 9 days. Flow cytometry was performed using a BD LSRFortessa, and flow cytometry data were analyzed using Modifit software. Collected BMSCs (800 rpm/min) were washed once with 1 mL phosphate-buffered saline (PBS), followed by the addition of 500 μL PBS containing 50 μg/mL ethidium bromide (PI) and 100 μg/mL RNase A; they were then incubated at 4 °C avoiding light for 20 min. Under a standard program, the excitation wavelength was 488 nm, and 1000–2000 cells were counted.

### Cell migration assay in vitro

The chemotaxis of macrophages was measured in a 24-well transwell culture chamber with an 8.0-μm pore polycarbonate membrane insert (Corning, USA). The control group, CS-N group, and CS-C group were placed in the lower chamber to investigate the chemotactic capacity of macrophages. Macrophages (RAW 264.7, BNCC) were seeded at 10^6^ per well in the upper chamber. After co-incubation for 24 and 48 h, the polycarbonate membrane was fixed in 4% paraformaldehyde and then stained with crystal violet solution. The number of migrated macrophages was counted with an optical microscope (Mingmei, Shenzhen, China). Five views of each sample were counted, and each group was evaluated four times.

### AMD3100 injection experiment

AMD3100 (Sigma-Aldrich, St Louis, MO) was subcutaneously injected to the wound area of receptor mice; 8 mg of AMD3100 per kilogram of body weight per day.

### Statistical analyses

Statistical analyses were performed using a statistical software package (GraphPad Prism 7.0). The unpaired two-tailed *t* test or one-way analysis of variance (ANOVA) were used to assess statistical significance. *P* values of 0.05 or less were considered significant.

## Results

### Characterization of the BMSC sheet

**Fig. 1:**
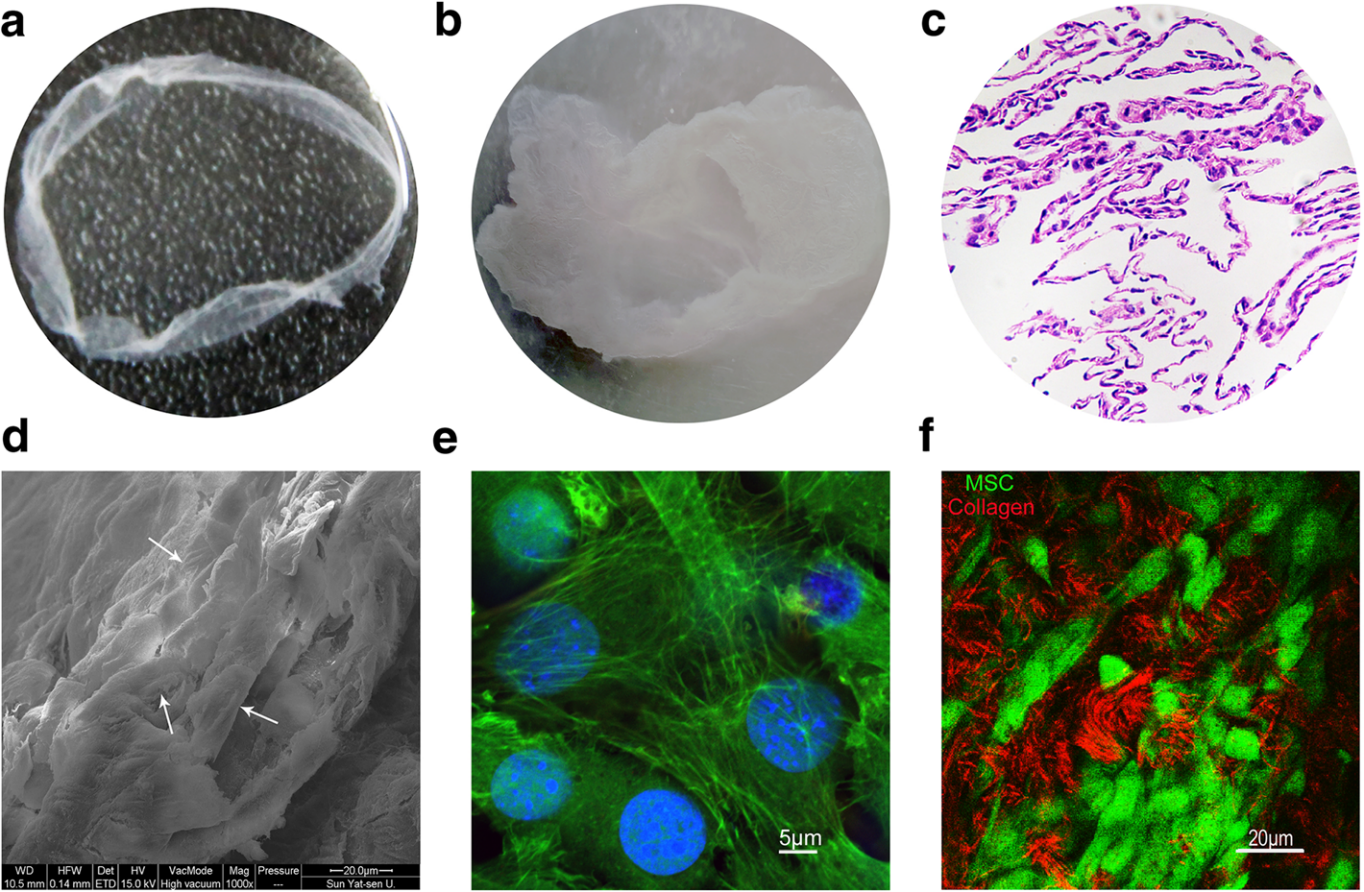
Characterization of the BMSC sheet. **a** The appearance of the BMSC sheet (6 cm in diameter). **b** Stereomicroscope image of the BMSC sheet (5×). **c** H&E staining of BMSC sheets which contained many layers of cells. **d** Scanning electron microscope image of the BMSC sheet; the arrows point to mesenchymal stem cells (MSCs) (1 kx, 20 μm). **e** Fluorescence microscope image of the BMSC sheet; *green* and *blue* show the cytoskeleton of GFP^+^ BMSCs and cell nuclei, respectively (63×, 5 μm). **f** Second harmonic imaging (SHG) image of the BMSC sheet; *red* and *green* represent collagen and cells, respectively (40×, 20 μm)

Third passage BMSCs were cultured in six-well plates with OriCell™ mouse BMSC Growth Medium supplemented with 0.5 μM curcumin and 100 mg/mL vitamin C. Up to the twelfth day, a white layer of cell membrane was observed (Fig. 1a). The macroscopic shape of this cell sheet was observed using a stereomicroscope (Fig. 1b) and exhibited a certain thickness and flexibility. H&E staining revealed that the cell aggregate in the curcumin-stimulated group (CS-C) was a membranous structure composed of collagen containing buried BMSCs (Fig. 1c). The SEM image revealed numerous GFP^+^ BMSCs in the sheet, which stacked together with extension of the culture time (Fig. 1d). These BMSCs presented spindles under green fluorescence using a confocal microscope (Fig. 1e). The special structure of the BMSC sheet was demonstrated by SHG, in which many BMSC layers surrounded bundles of collagen and some BMSCs were on the collagen surface, some were under the collagen, and some were interspersed between the collagen (Fig. 1f).

### Influence of curcumin on BMSC proliferation activity

**Fig. 2:**
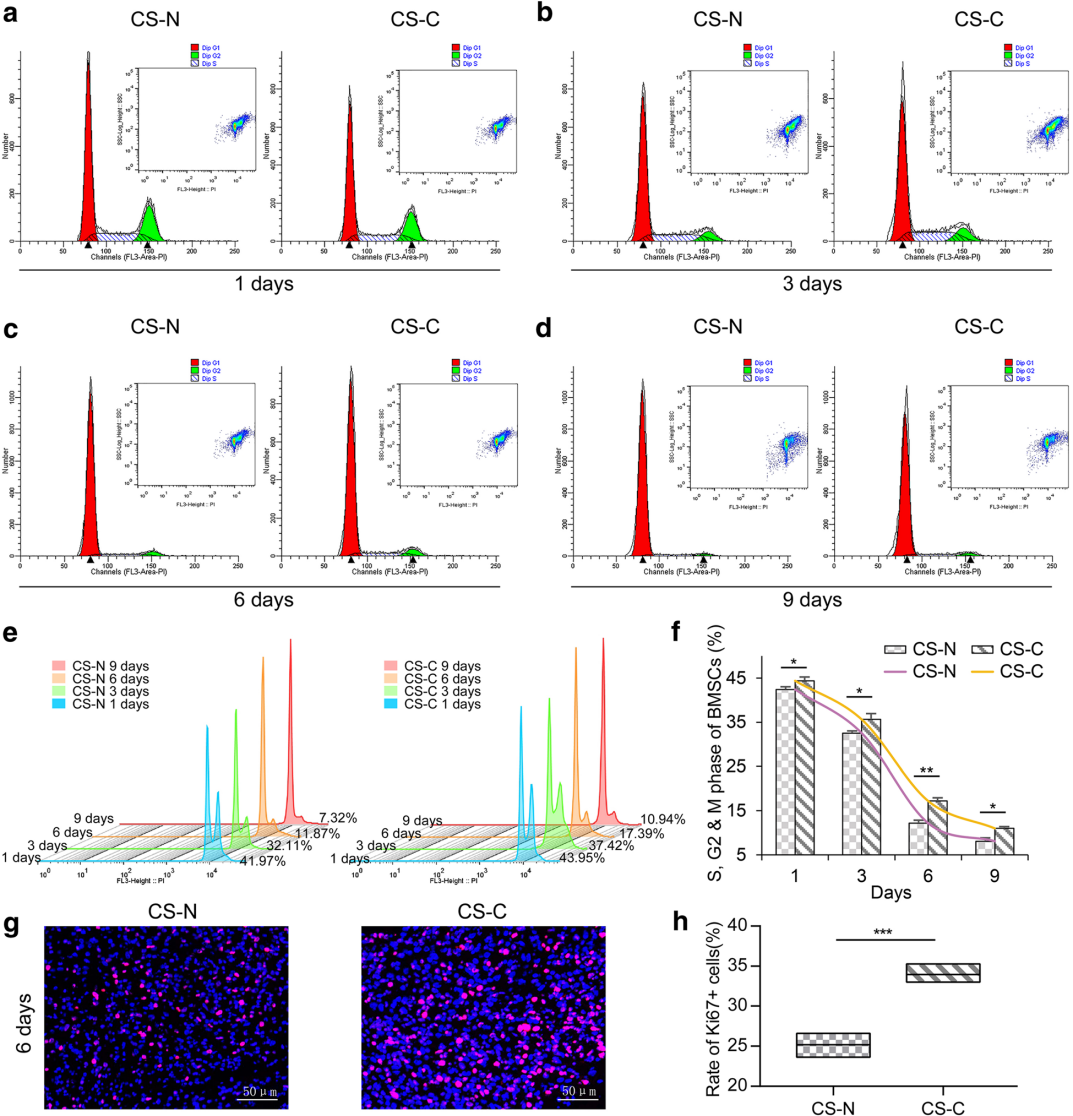
Influence of curcumin on bone marrow-derived mesenchymal stem cell (BMSC) proliferation activity. BMSCs treated with curcumin at a concentration of 0.5 μM (CS-C) and without curcumin (CS-N) for 1 day (**a**), 3 days (**b**), 6 days (**c**), and 9 days (**d**), respectively. The cell cycle was determined by flow cytometry. **e** Flow cytometry to assess the cell cycle at the indicated intervals (*n* = 3). **f** Quantification of the number of MSCs at the S, G2, and M phase. **g** Immunofluorescence staining of the proliferation of cells with Ki67 in the CS-N and CS-C groups (cultured for 6 days). **h** Quantification of the rate of Ki67-positive cells in CS-N and CS-C groups (*n* = 4). Student’s *t* test: *P < 0.05, **P < 0.01, ***P < 0.001

As discussed above, small molecules have a strong impact on cellular activity. The activity of BMSCs was also greatly enhanced after the application of 0.5 μM curcumin (Fig. 2a–d). Because the formation of BMSC sheets requires 12 days, the growth rate of the cells gradually decreased during the process. However, this decline could be relieved by curcumin (Fig. 2e). A greater number of BMSCs were in the S, G2, and M period after curcumin treatment. Additionally, the number of active cells increased significantly by 4.63%, 9.51%, 41.09%, and 35.78%, respectively, after 1, 3, 6, and 9 days of exposure to curcumin (Fig. 2f). Also, the CS-C sheet showed increased expression of the cell proliferation marker Ki67 than that of the CS-N sheet, suggesting a promotion ability of curcumin on BMSC proliferation (Fig. 2g, h).

### Engraftment of GFP^+^ BMSCs during wound healing

**Fig. 3:**
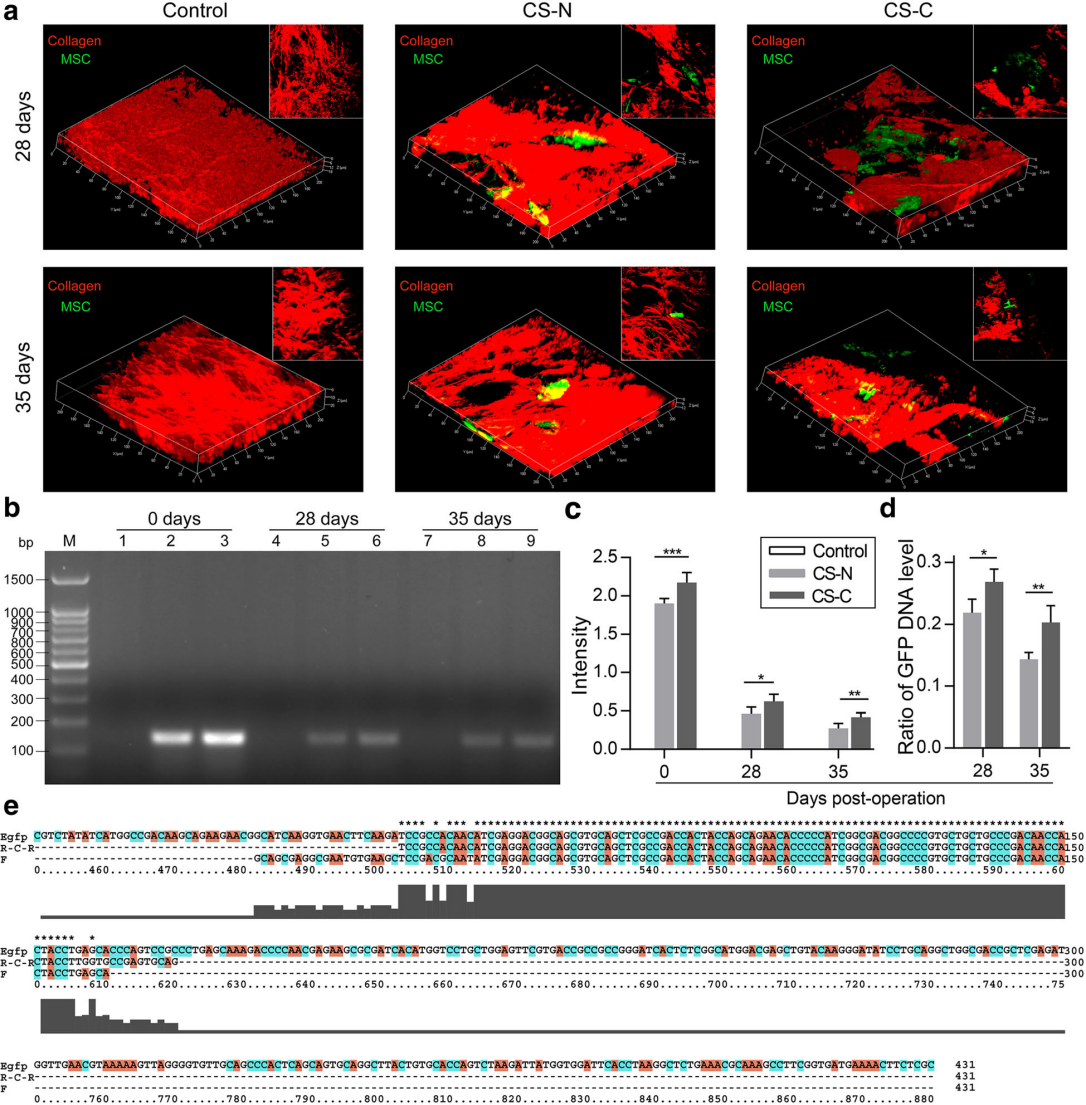
Engraftment of GFP^+^ BMSCs during wound healing. **a** Second harmonic imaging (SHG) of control (left), BMSC sheets induced without curcumin (CS-N; middle), and BMSC sheets induced by curcumin (CS-C; right) transplanted into the wound site at 28 days and 35 days. Green and red indicate GFP^+^ mesenchymal stem cells (MSCs) and collagen, respectively. **b** The contents of GFP^+^ BMSCs represented by DNA agarose gel electrophoresis; each lane shows control, CS-N, and CS-C, respectively, at 0 days (lanes 1, 2, 3), 28 days (lanes 4, 5, 6), and 35 days (lanes 7, 8, 9). **c** Relative quantitative analysis of the content of GFP DNA (*n* = 4 mice/group). **d** Ratio of green fluorescent protein (GFP) DNA level at 28 days and 35 days to 0 days. **e** Sequence alignment results for the amplified products with GFP sequences. Student’s *t* test (**d**) and ANOVA (**c**): *P < 0.05, **P < 0.01, ***P < 0.001

To detect the plasticity of GFP^+^ BMSCs, the healing wound tissue was measured after 28 and 35 days by TPF-SHG. Incredibly, GFP^+^ BMSCs could be detected up to 35 days in the CS-N and CS-C group (Fig. 3a). To quantify the number of surviving cells, we extracted DNA from skin that had been repaired for 0, 28, and 35 days. In Fig. 1b, lanes 1, 2, and 3 represent the control group, CS-N group, and CS-C group, respectively, at 0 days; lanes 4, 5, and 6 represent the three groups at 28 days, and lanes 7, 8, and 9 represent the three groups at 35 days. At 42 days, BMSCs were hardly detected in all groups (data not shown). The results showed that GFP DNA in the CS-C group was increased 1.17-fold compared to the CS-N group at 0 days post-operation. However, after 28 days and 35 days of engraftment, the GFP DNA was 1.34-fold and 1.53-fold increased, respectively, in the CS-C group compared to the CS-N group. Furthermore, the ratios of GFP^+^ BMSC content at 28 days post-operation and 35 days post-operation compared with 0 days post-operation were 0.269 and 0.203 in the CS-C group and 0.219 and 0.143 in the CS-N group, respectively (Fig. 3d), which meant that there was a lower decreasing rate in the content of BMSCs in CS-C than that in CS-N. At 0, 28, and 35 days, the target bands of electrophoresis were excised for sequence alignment in both the forward and reverse directions. The product sequence was approximately 135 bp. The matching rate of the two sequences was very high (Fig. 3e). Overall, GFP^+^ BMSCs in the CS-C group showed improved engraftment and survival before 6 weeks than did those in the CS-N group.

### Effect of curcumin on ECM secreted by the BMSC sheets

**Fig. 4:**
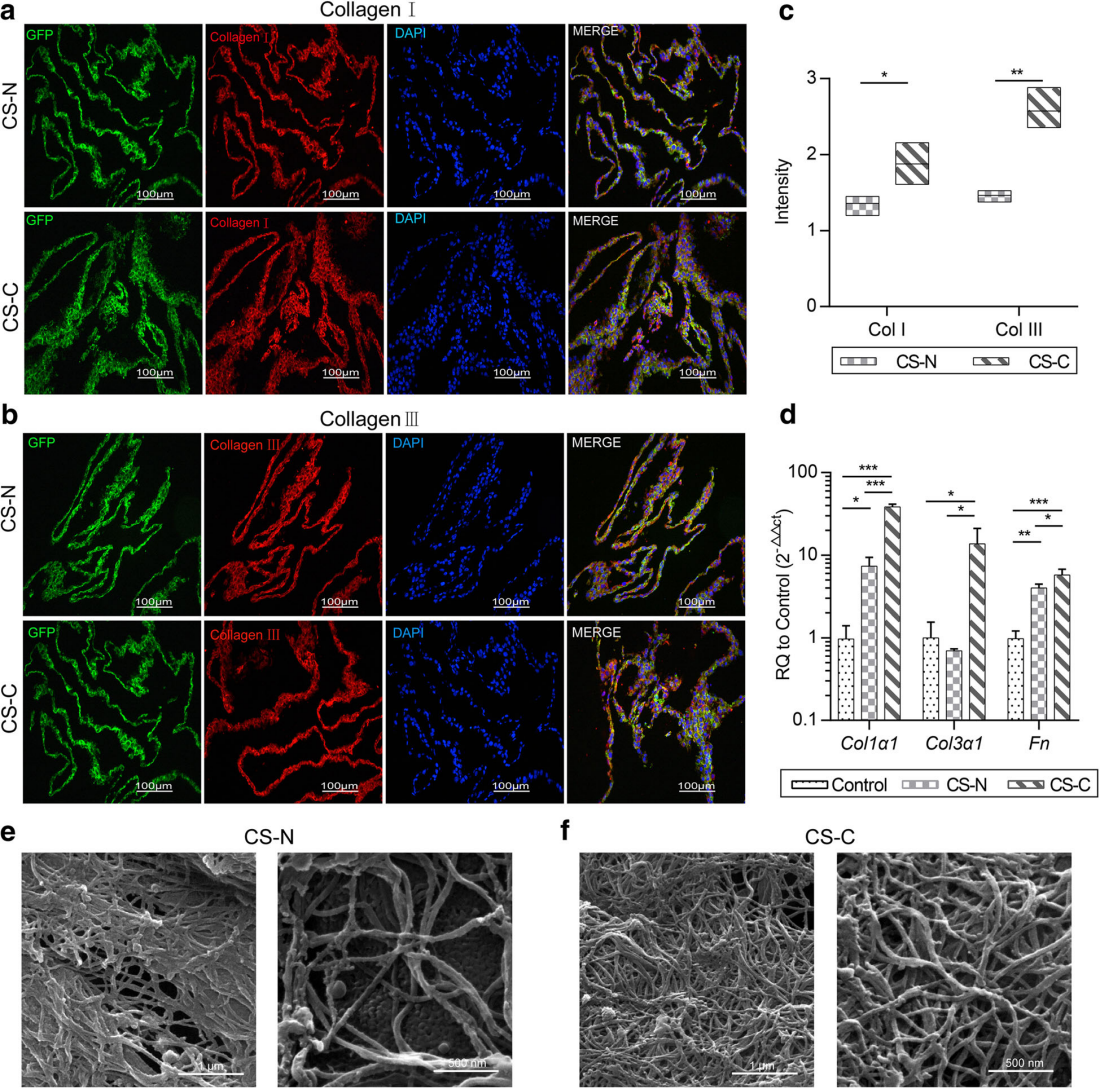
Effect of curcumin on the BMSC sheets. **a,b** Immunohistochemical staining of the BMSC sheets induced without curcumin (CS-N) and BMSC sheets induced by curcumin (CS-C); *green*, *red*, and *blue* represent green fluorescent protein (GFP)^+^ cells, collagen I (**a**)/collagen III (**b**), and nuclei, respectively (20×, 100 μm) (*n* = 4). **c** Content variation in collagen (Col) I and collagen III in the CS-N group and CS-C group. **d** RT-PCR results for gene expression in the extracellular matrix, including *col1α1*, *col3α1*, and *Fn*. SEM image of the **e** CS-N group and **f** CS-C group (left is 20 kx, 1 μm, right is 40 kx, 500 nm). Student’s *t* test and ANOVA: *P < 0.05, **P < 0.01, ***P < 0.001

BMSC sheets use the ECM secreted during the culture period as an endogenous scaffold, which is important for wound healing. The results of immunofluorescence showed that the secretion of types I and III collagen was increased in cell sheets cultured with curcumin (Fig. 4a, b). The growth of type III collagen in the CS-C group was almost double that in the CS-N group (Fig. 4a–c). ECM-related genes, including collagen type I, collagen type III, and fibronectin (FN), were determined by RT-PCR. The PCR results were consistent with the results of immunofluorescence. A higher expression of genes, including *col1α1*, *col3α1*, and *Fn*, was detected in the CS-C group compared with the CS-N group and control group (BMSCs cultured in a culture bottle for 2 days) (Fig. 4d). To explore the ultrastructure of this endogenous scaffold, we compared the two scaffolds using an SEM. The cell sheets cultured with curcumin clearly had a more complete and thicker collagen structure (Fig. 4e, f).

### CS-C recruits macrophage (M) and T cells in early repair stages

**Fig. 5:**
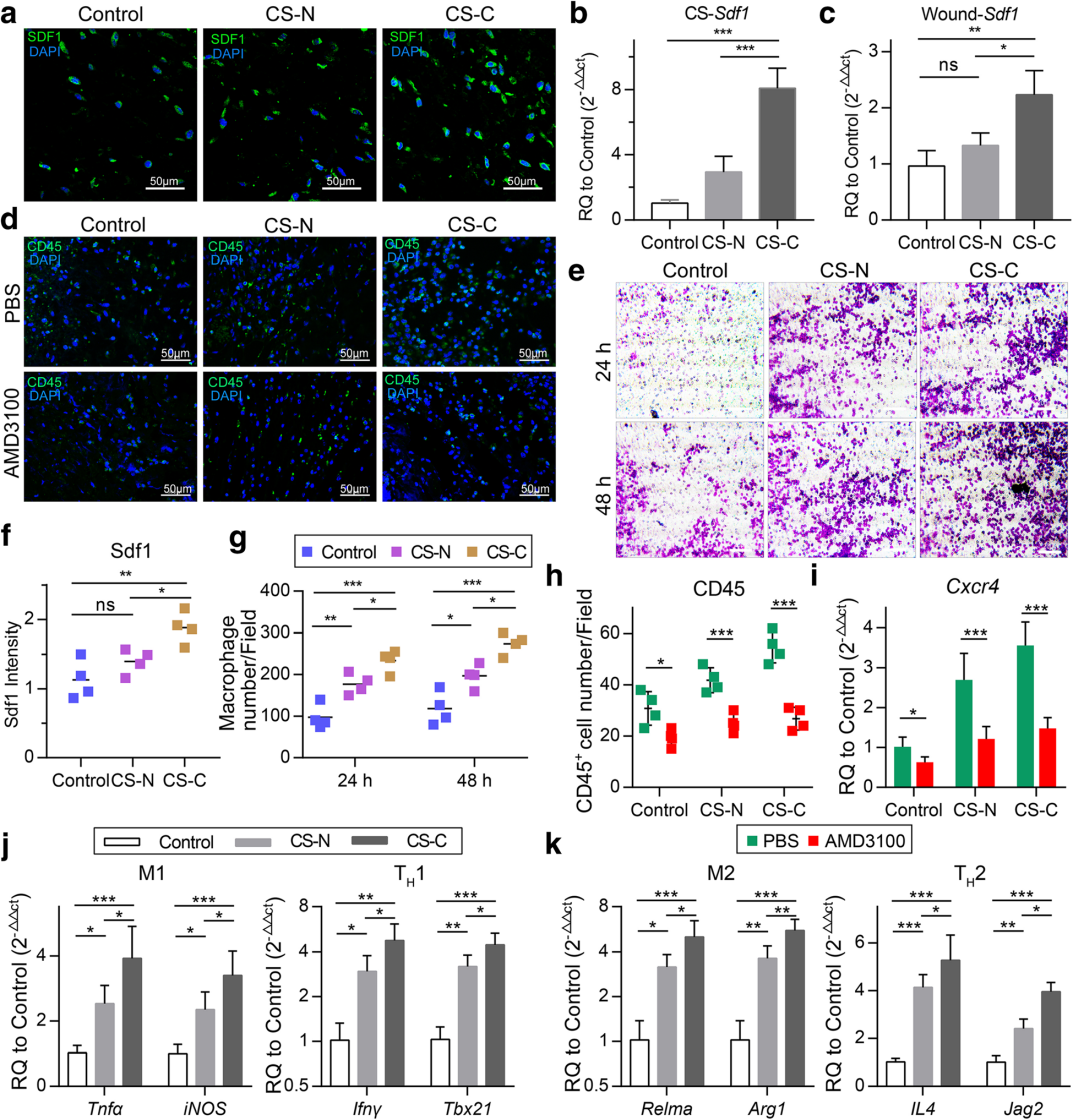
CS-C recruits macrophages and T cells in early repair stages dependent on SDF1. **a** Immunofluorescence staining of stromal cell-derived factor 1 (SDF1) in the wound area in the control, BMSC sheets induced without curcumin (CS-N), and BMSC sheets induced by curcumin (CS-C) groups at 7 days post-operation (40×, 50 μm) (*n* = 4 mice/group). **b** Gene expression of SDF1 in the control, CS-N, and CS-C groups in vitro. **c** Gene expression of SDF1 in the wound area at 7 days post-operation. **d** Immunofluorescence staining of CD45^+^ leukocytes in the wound area in the control, CS-N, and CS-C groups at 7 days post-operation with AMD3100 or without (phosphate-buffered saline (PBS) instead of AMD3100) (40×, 50 μm) (*n* = 4 mice/group). **e** Macrophage chemotaxis in the control, CS-N, and CS-C groups at 24 and 48 h (20×, 50 μm). **f** Quantification of the SDF1 intensity at 7 days-post-operation. **g** Quantification of the number of migrated macrophages. **h** Quantification of the number of CD45^+^ cells at 7 days post-operation. **i** Gene expression of *Cxcr4* in the control, CS-N, and CS-C groups at 7 days post-operation with or without AMD3100 (*n* = 5 mice/group). **j** Expression levels of *Tnfα*, *iNOS* (signature genes of M1 macrophages), and *Ifnγ*, *Tbx21* (signature genes of T_H_1 cells) by RT-PCR at 7 days-post-operation. **k** Expression levels of Relmα, *Arg1* (signature genes of M2 macrophages), and *IL4*, *Jag2* (signature genes of T_H_2cells) by RT-PCR (*n* = 5 mice/group). ANOVA: *P < 0.05, **P < 0.01, ***P < 0.001

To explore the effects of CS-C on the immune response, we examined the immune environment around the wound site. First, we found that the gene expression of SDF1 in the curcumin treatment group was much higher than that in the no curcumin treatment group during the process of cell sheet culture (Fig. 5b). Also, this effect lasted until the sheet was transplanted into the wound at 7 days post-operation; the immunofluorescence images accompanying the results of PCR demonstrate this (Fig. 5a, c, f). SDF1 is a molecule that possesses potent chemotactic activity for leukocytes to participate in the inflammatory response on wound healing. Indeed, in our study, CD45^+^ leukocytes infiltration varied greatly between groups at 7 days post-operation. CD45^+^ leukocyte infiltration in the CS-C group was twice that in the control group and 1.5 times that in the CS-N group (Fig. 5d), in favor of the detection and removal of germs. To further confirm whether CS-C had a recruitment effect on leukocytes, we selected macrophages to conduct in vitro migration experiments. The results showed that after 24 and 48 h CS-C did have a recruitment effect on macrophages, and more macrophages were recruited over time (Fig. 5e, g). To study whether SDF1 was the dominant factor inducing the leukocyte recruitment, we used AMD3100 to verify the role of SDF1. AMD3100 is the antagonist of CXCR4, a receptor specific for SDF1. The results showed that the gene expression of CXCR4 was significantly reduced with AMD3100 (Fig. 5i). CD45^+^ leukocyte infiltration also demonstrated a large reduction with the use of AMD3100 at 7 days post-operation (Fig. 5d, h), suggesting a direct role for SDF1 in recruiting leukocytes. As reported above, macrophages have two different types of function: pro-inflammation M1 and tissue-repairing M2, which correspond to T_H_1 and T_H_2 cells. In the PCR experiment in vivo, the expression of signature genes of M1 macrophages (*Tnfα*, *iNOS*), M2 macrophages (*Relmα*, *Arg1*), T_H_1 cells (*Ifnγ*, *Tbx21*), and T_H_2 cells (*IL4*, *Jag2*) (Jag2 encodes the Notch ligand Jagged2, which helps T_H_2 differentiation) was significantly increased in the CS-C group compared with the control group and CS-N group (Fig. 5j, k). Thus, CS-C enhanced the immune response when first transplanted into the wound.

### CS-C accelerates type I immune reaction regression and M2 macrophage polarization

**Fig. 6:**
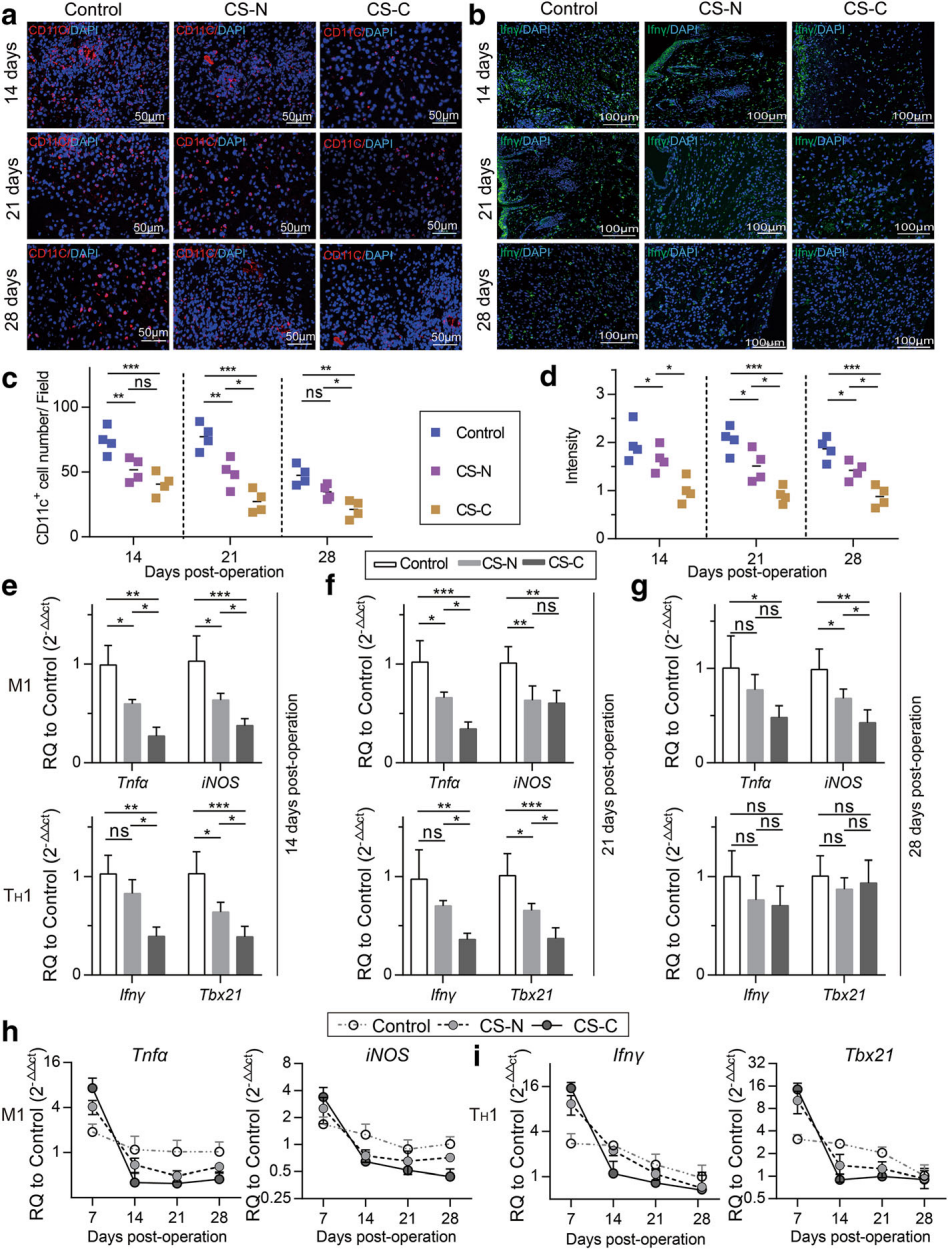
CS-C accelerated type I immune reaction regression. **a** Immunofluorescence staining of CD11c^+^ M1 macrophages in the control, BMSC sheets induced without curcumin (CS-N), and BMSC sheets induced by curcumin (CS-C) groups at 14, 21, and 28 days after treatment. Red and blue represent CD11c^+^ M1 macrophages and nuclei, respectively (20×, 50 μm) (*n* = 4 mice/group). **b** Immunofluorescence staining of interferon (IFN)γ at 14, 21, and 28 days. Green and blue represent T helper 1 (T_H_1) cells and nuclei, respectively (bar = 100 μm) (*n* = 4 mice/group). Quantification of **c** the number of M1 macrophages and **d** T_H_1 cells, respectively. Expression levels of *Tnfα* and *iNOS* (signature genes of M1 macrophages) and *Ifnγ* and *Tbx21* (signature genes of T_H_1 cells) by RT-PCR at 14 (**e**), 21 (**f**), and 28 days (**g**) after transplantation with saline, CS-N, and CS-C (*n* = 5 mice/group). M1 (**h**) and T_H_1 (**i**) signature gene expression during the whole experimental period for the control, CS-N, and CS-C groups. ANOVA: *P < 0.05, **P < 0.01, ***P < 0.001. ns not significant

After 7 days post-operation, there were tremendous changes in the immune environment. The number of pro-inflammatory M1 macrophages and associated T_H_1 cells declined dramatically. The results of immunofluorescence showed that the number of M1 macrophages and T_H_1 cells as characterized by CD11c and IFNγ in the CS-C group was much lower than that in the CS-N and control groups at 14, 21, and 28 days post-operation (Fig. 6a–d). Furthermore, there was a significant decrease in *Tnfα* and *iNOS* (signature genes of M1 macrophages) and *Ifnγ* and *Tbx21* (signature genes of T_H_1cells) mRNA between the CS-C group and the CS-N and control groups at 14 and 21 days post-operation, but no significant difference at 28 days post-operation (Fig. 6e–g). The above results show that the CS-C group has a shorter type I immune response and can accelerate type I immune reaction regression. The general trend is that the M1 macrophages, T_H_1 cells, increase dramatically by 7 days, decline after 14 days, and then moderate by 28 days (Fig. 6h, i).

**Fig. 7:**
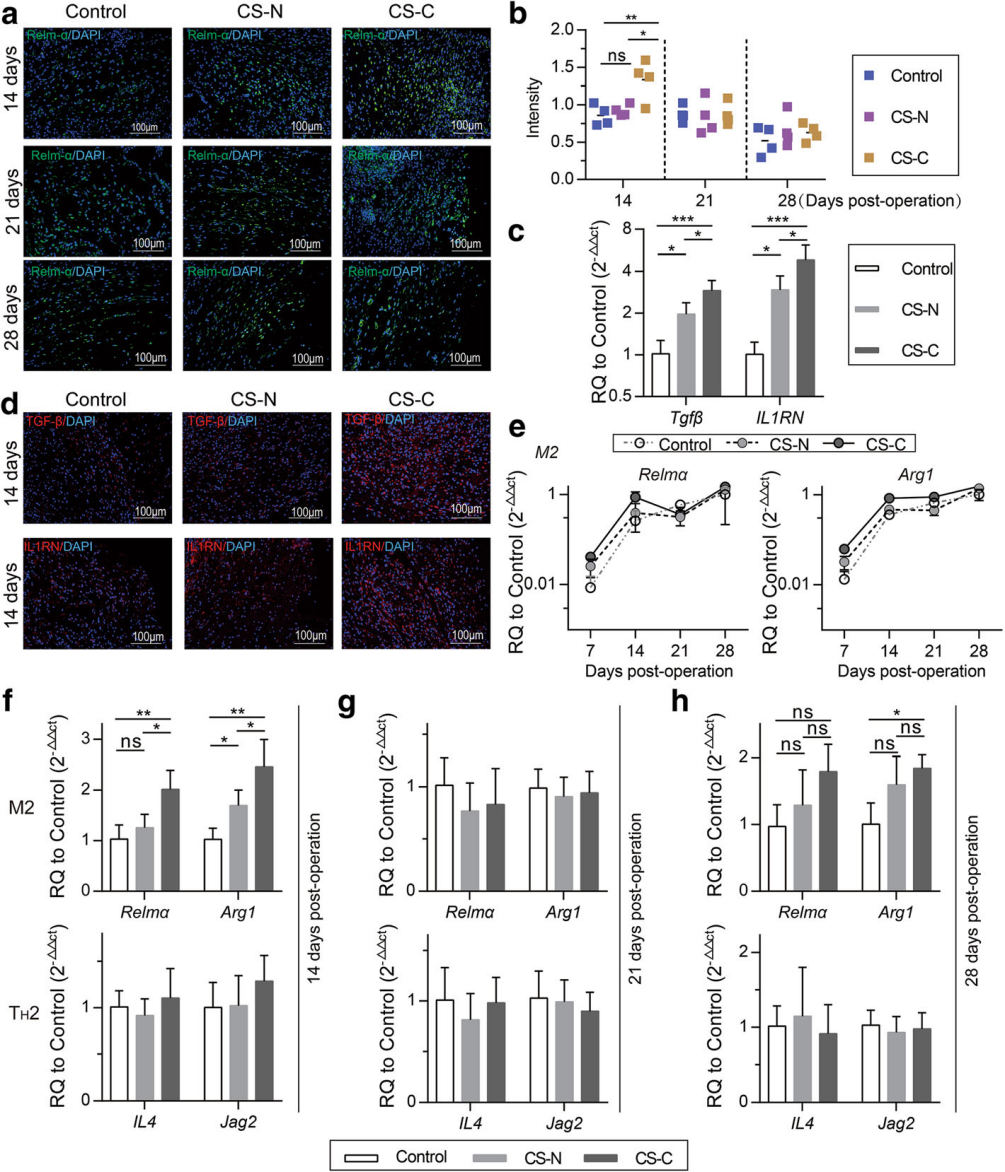
CS-C accelerated M2 macrophage polarization. **a** Immunofluorescence staining of Relmα in the control, BMSC sheets induced without curcumin (CS-N), and BMSC sheets induced by curcumin (CS-C) groups at 14, 21, and 28 days after treatment. Red and blue represent Relmα and nuclei, respectively (20×, 100 μm) (*n* = 4 mice/group). **b** Quantification of the Relmα intensity. **c** Expression levels of *Tgfβ* and *IL1RN* by RT-PCR at 14 days post-operation (*n* = 5 mice/group). **d** Immunofluorescence staining of Tgfβ^+^ (top panels) and IL1RN^+^ (bottom panels) cells in the three groups at 14 days post-operation. Red represents Tgfβ and IL1RN, and blue represents nuclei (20×, 100 μm) (*n* = 4 mice/group). **e** M2 signature gene expression during the whole experimental period for the control, CS-N, and CS-C groups. Expression levels of *Relmα* and *Arg1* (signature genes of M2 macrophages) and *IL4* and *Jag2* (signature genes of T_H_2 cells) by RT-PCR at 14 (**f**), 21 (**g**), and 28 days (**h**) after transplantation with saline, CS-N, and CS-C (*n* = 5 mice/group). ANOVA: *P < 0.05, **P < 0.01, ***P < 0.001. ns not significant

On the other hand, an increasing number of M2 macrophages were activated. The mRNA level of the M2 macrophages, marked by *Relmα* and *Arg1*, tended to be increased in the CS-C group at 14 days post-operation and there was no significant difference between the three groups at 21 or 28 days post-operation (Fig. 7f–g). The results of Relmα^+^ M2 macrophage immunofluorescence also confirmed this (Fig. 7a, b). More M2 macrophages in the CS-C group were polarized during 7 to 14 days post-operation compared to the situation at 7 days post-operation (Fig. 7e). Furthermore, immunosuppressive cytokines secreted by BMSCs, such as TGFβ and IL1RN, were shown to promote the polarization of M2 macrophages. The mRNA levels of *Tgfβ*, *IL1RN* and Tgfβ^+^, IL1RN^+^ immunofluorescence increased in the CS-C group at 14 days post-operation compared to the CS-N and control groups (Fig. 7c, d). Therefore, the CS-C group demonstrated the capability of recruiting more tissue-repairing immune cells to promote wound regeneration in the later stages of wound repair. At the same time, it effectively prevented chronic inflammation.

### CS-C promotes skin reconstruction

**Fig. 8:**
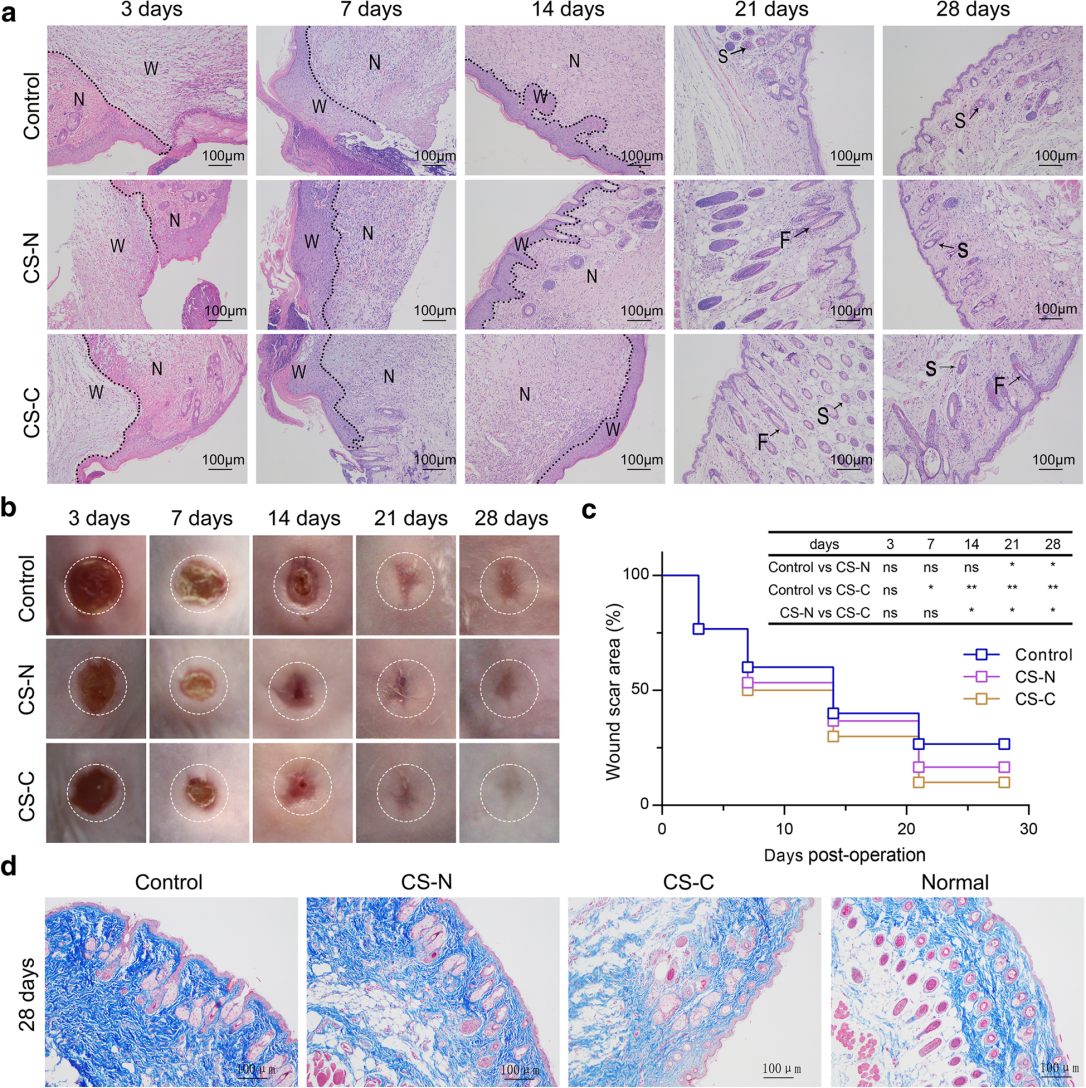
CS-C promotes skin reconstruction. **a** Immunohistochemical staining (10×, 100 μm) and **b** macroscopic observation of the wound areas in the control, BMSC sheets induced without curcumin (CS-N), and BMSC sheets induced by curcumin (CS-C) groups at days 3, 7, 14, 21, and 28 (*n* = 5 mice/group). **c** Statistical analysis of wound closure rate in continuous time (3, 7, 14, 21, and 28 days). **d** Masson’s trichrome staining (10×, 100 μm) of healing wounds in the control group, CS-N group, CS-C group, and normal group at 28 days post-operation. ANOVA: *P < 0.05, **P < 0.01, ***P < 0.001. F hair follicle, N normal site, ns not significant, S sebaceous gland, W wound site

Mice were euthanized at 3, 7, 14, 21, and 28 days post-operation to evaluate whether the wound was repaired better after CS-C treatment. Skin samples were used to observe what changes had occurred in the wound tissue during the wound healing process. First, we observed that the wound was fully closed after about 14 days with a small and obscure scar found after 28 days in the CS-C group. However, in the control and CS-N groups, the wound was only almost closed at 21 days post-operation, with a scab covering. Furthermore, a clear, over fibrotic scar could be seen in the later stages of repair of the CS-N and control groups (Fig. 8b, c). To further analyze the details of the wound healing, we stained 3, 7, 14, 21, and 28 days post-operation wound samples with H&E. The CS-C group began to form new epidermis at 7 days post-operation, but neoepidermis was only noted at 14 days post-operation for the control and CS-N groups. At 28 days post-operation, stratified epidermis structures, including hair follicles and sweat glands, could be observed in the CS-C group. On the other hand, the epidermal thickness of the CS-C group at 28 days post-operation was more like that of normal skin tissue than the control or CS-N groups (Fig. 8a). Masson trichrome staining indicated that the CS-C group had mature bundles of collagen deposition in a certain direction and more dense blood vessels, more like the normal group, while the control and CS-N groups has disorganized collagen (Fig. 8d).

## Discussion

BMSC sheet technology has been studied for applications over recent years, and one of its major advantages is that it could avoid the damage of enzyme digestion to preserve better activity of the BMSCs. BMSCs have a beneficial effect on cutaneous wound healing and skin regeneration in many different ways, including differentiation, endocrine, and paracrine effects, as well as the most important immune response modulatory capability [31, 32]. BMSCs secrete diverse cytokines and growth factors that have anti-fibrotic properties to minimize the formation of scar tissue, such as hepatocyte growth factor (HGF), which has been shown to suppress overexpression of collagens and increase MMP-1 and MMP-13 expression in fibroblasts [33, 34]. Moreover, BMSCs communicate with the inflammatory microenvironment to initiate their immunomodulatory functions. When stimulated by IFNγ, TNFα, or IL-1α, BMSCs exhibit immunosuppression, inhibiting T-cell proliferation and differentiation into T_H_1 cells [35]. Other studies show that BMSCs are effective for converting macrophages from the pro-inflammatory M1 type to the anti-inflammatory M2 type [14, 15].

With this mind, strategies to mediate BMSCs to produce a desirable BMSC sheet with further enhancement of the proliferation of BMSCs, and to guide the expression of suitable wound repair factors thus inducing a proper immune response to promote wound healing, are key points of this study. It is wise to regulate BMSCs, which are beneficial for skin regeneration, via epigenetic modifications. Some suitable small molecules that can be easily synthesized, preserved, and standardized are the first choice [36]. Studies have confirmed that the functional ingredients in certain Chinese herbs can influence cell behavior [37] and further improve BMSC sheets to promote bone formation in metaphyseal defects [38]. Curcumin is a natural polyphenolic substance extracted from zingiberaceae roots, and has been shown to have anti-inflammatory and anti-fibrosis effects in cutaneous wound healing. Therefore, in this study, we initiated a modified epigenetic strategy using curcumin to rejuvenate BMSCs and to improve the functional BMSC sheet to promote cutaneous wound healing. Modified epigenetics refers to those variations of epigenetic components that result in changes in specific chromatin architecture that activate or deactivate expression of the regulatory genes, and finally impose influence on cell fate determination but without changing DNA sequences [39]. In our study, after being stimulated by curcumin, BMSCs transcribed more SDF1 to recruit immune cells at 7 days post-operation, and more immunosuppressive factors such as TGFβ and IL1RN were secreted by BMSCs at 14 days post-operation to suppress the type I immune response and promote M2 polarization; therefore, the application of curcumin was thought to be a modified epigenetic strategy for BMSCs.

The therapeutically relevant delivery and survival rate of BMSCs grafted to the wound area are extremely important parameters for wound regeneration, and determines for how many and how long the BMSCs exert their pro-healing effects [40]. However, in most studies, such as in the system of BMSC administration alone, the delivered number of BMSCs for engraftment is poor or the engrafted BMSCs tend to be short-lived in the wound area, surviving around 14 days [41]. However, after implantation of the curcumin-mediated BMSC sheet, both TPF/SHG imaging and DNA amplification demonstrated that GFP^+^ BMSCs could still be detected up to 35 days post-operation. Furthermore, BMSCs in the CS-C group showed better engraftment than those in the CS-N group. More BMSCs survived and, of these, more BMSCs were in the cell replication period of S, G2, or M phase after treatment with curcumin, as shown in Fig. 2f, suggesting that curcumin significantly enhanced the proliferation of BMSCs. Previous research by ourselves and others have shown that BMSCs affect other cells primarily through paracrine effects to participate in the process of tissue repair and immune regulation [30]. Greater numbers and longer survival rates ensured that the BMSCs secreted more cytokines to promote wound healing by enhancing the immunomodulatory ability of the above-described BMSCs. Curcumin amplified the delivery and efficacy of BMSCs and thus could reinforce its pro-healing functions by promoting vascular endothelial cell proliferation to increase angiogenesis, regulating macrophage and T-cell functions to avoid chronic inflammation, as well as producing anti-fibrotic factors to reduce scar formation [42, 43]. Moreover, secretion of SDF1 by the BMSC sheet was greatly increased after curcumin treatment as shown in Fig. 5b. In addition to being able to recruit large number of leukocytes, SDF1 was sufficient to enhance endogenous BMSC recruitment to the injury site [44]. In our study, endogenous BMSCs may have also been recruited to promote wound healing; however, this possibility requires further research.

It is clear that immune cells are important regulators of regeneration, not only because of their ability to prevent infection and clear necrotic tissue but also because of their powerful influence in shaping the microenvironment during repair and development. The persistent presence of pro-inflammatory factors such as TNFα and IFNγ, among others, secreted by T_H_1 and M1 macrophages may result in extensive secondary damage and chronic inflammation, and can compromise the function of tissues [45]. However, there is also evidence to indicate that the existence of IFNγ, TNFα, and IL-1β activate the immunomodulatory phenotype of BMSCs [46]. In our study, the abundance of SDF1 produced by CS-C led to a mass migration of leukocytes; among these, T_H_1 cells and associated pro-inflammatory M1 macrophages demonstrated an increase at 7 days post-operation. The immunosuppressive phenotype of BMSCs might then be activated by the secreted pro-inflammatory factors (IFNγ, TNFα) via those leukocytes and release several factors relevant to the immunosuppressive function, including TGFβ and IL1RN [47]. It is worth noting that IL1RN and TGFβ have been shown to promote the differentiation of M2 macrophages [48, 49]. To confirm this, increased expression of TGFβ and IL1RN in the CS-C group at 14 days post-operation was seen in our study (Fig. 7d). The number of T_H_1 cells and M1 macrophages was subsequently found to be sharply decreased in the CS-C group at 14 days post-operation (Fig. 6h, i), suggesting a shorter type I immune response here, which is thought to be critical for improving the survival rate of BMSCs and avoiding chronic inflammation [4, 50]. Also, recent studies have suggested that the type II immune response can improve inflammation-mediated tissue damage and induce repair in missing tissue [51], while a persistent presence of excessive M2 macrophages or T_H_2 cell activities could unavoidably cause fibrosis [52–54]. In the CS-C group in our study, during the early stage of repair, there was a strong trend towards T_H_2 activation (0–7 days) and M2 polarization (7–14 days) and, fortunately, at the later stage (after 14 days), the number of M2 macrophages fell (Fig. 7e). This is a favorable immune response which is generally believed to produce anti-inflammatory effects and can improve wound repair [55]. The Masson’s trichrome staining in Fig. 8d showed that the rebuilt tissue collagen in the CS-C group was more like normal tissue in content and organization than that in the CS-N and control groups. In addition, M2 macrophages, if in a substantial number, have been shown to have an inhibitory function on the activation of M1 macrophages and T_H_1 cells [56]. The existence of a large number of M2 macrophages during days 7–14 of repair may explain the decline in T_H_1 cells and M1 macrophages in the later period (after 14 days) in our study, as shown in Figs. 6h, i, and 7e. The curcumin-treated cell sheet (CS-C), therefore, has been shown to have a powerful ability to create a pro-regenerative microenvironment at the site of implantation.

ECM provides cells with a dynamic niche and influences the cell immune microenvironment as a scaffold. Biological scaffolds sourced from ECM are thought to be beneficial to tissue repair [57, 58] and have been successfully used for tissue reconstruction in skin, skeletal muscle, the esophagus, and the lower urinary tract [59–62]. Recent research has demonstrated that ECM scaffolds possess the ability to alter the default wound healing response from pro-inflammatory towards pro-regenerative [15]. We noticed in our study that curcumin promoted high secretion levels of Fn, which mediates cell-matrix adhesion and enhances the contents and the ratio of collagen III and I; these constitute the main framework of the endogenous ECM in the BMSC sheet. Therefore, we are aware that curcumin alters the features of endogenous ECM in the BMSC sheet. Amino acids are believed to be in charge of the polarization of M2 macrophages in the immune response of wound healing. It is proposed that amino acids play a role via amino acid-sensing pathways (Lamtor1, v-ATPase, and mTORC1) where the amino acid is engulfed by macrophages and digested by their abundantly developed lysosomes. Sufficient amino acids and IL-4 integrate to the lysosomal adaptor protein Lamtor1, v-ATPase, and mTORC1, leading to production of 25-hydroxycholesterol and subsequent activation of liver X receptor (LXR), which ultimately results in polarization of M2 macrophages [7]. In our study, collagen in the endogenous ECM scaffold could be a possible rich source of amino acids, and the enhancement in collagen content in the ECM in the CS-C group could be the reason for better M2 macrophage polarization association. Also, it is well known that in fetal skin there is a process of scarless wound healing. This wound healing response of fetal skin is believed to be intrinsic to the fetal skin ECM rather than the fetal environment [63]. Unlike adult skin ECM which is primarily composed of collagen I, fetal ECM has high ratio of collagen III/I [64]. Our endogenous ECM scaffold induced by CS-C possesses properties that are more like that of fetal skin, with a high ratio of collagen III/I (Fig. 4d), and this may be helpful in reducing the effect of scar tissue in adult full-thickness cutaneous wound healing.

**Fig. 9:**
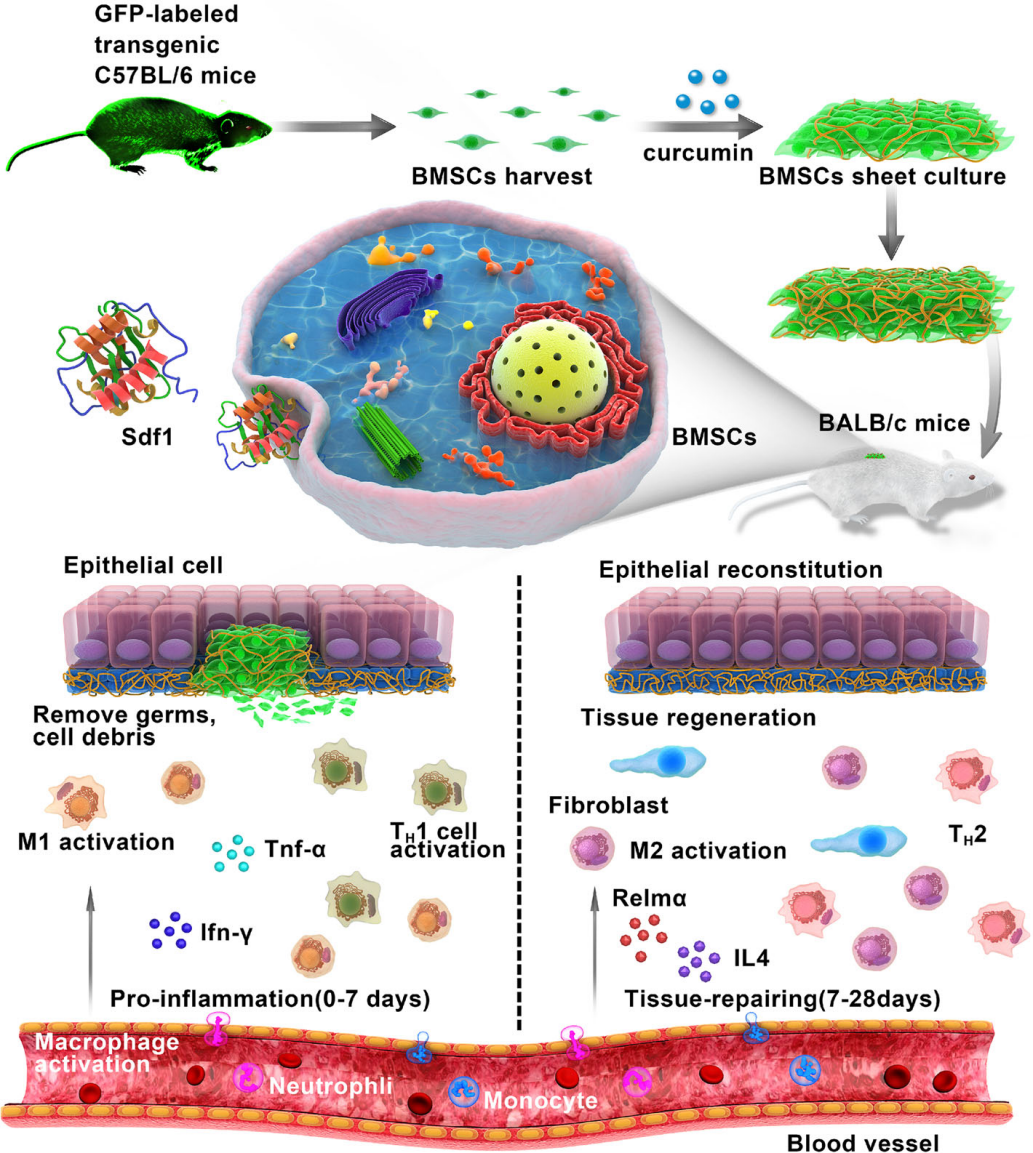
The CS-C-based immunomodulatory process in wound healing. Green fluorescent protein (GFP)^+^ bone marrow-derived stem cells (BMSCs) were harvested from transgenic C57BL/6 mice. BMSC sheets were prepared by culturing 1.5 × 10^5^ third passage cells on culture dishes for 12 days. The BMSC sheets were then transplanted into the skin wounds of the recipient mice. Once CS-C is applied to the wound site, it induces the secretion of various chemokines. Stromal cell-derived factor 1 (Sdf1) increases significantly, recruiting more leukocytes, such as macrophages (M) and T helper (T_H_) cells, to the wound area. Abundant type I immune cells of M1 macrophages and T_H_1 cells are activated at approximately 7 days of repair in the pro-inflammatory stage. The temporary pro-inflammatory response leads to the rapid removal of foreign pathogens. After 7 days post-operation, the typical type I immune response, the number of M1 macrophages and T_H_1 cells near the wound are greatly reduced; instead, there is an increase in type II immune response, as reflected in the number of anti-inflammatory M2 macrophages. Timely suppression of the type I immune reaction allows rapid tissue rebuilding, and the increase in type II anti-inflammatory M2 macrophages in the following stage is beneficial for tissue repairing. Through the transplantation of CS-C, rapid and effective skin wound healing occurs. Ifn interferon, IL interleukin, Tnf tumor necrosis factor

To summarize, we demonstrated that the Traditional Chinese Medicine curcumin is an effective regulator for altering the characteristics of the BMSC sheet. It not only allows BMSCs to present better proliferating activity and better engraftment, but it also produces a more suitable endogenously secreted ECM scaffold. Most importantly, this new type of biomaterial establishes an immune response that is favorable for adult cutaneous wound healing, which promotes repair of the cutaneous wound quickly and with improved quality. Figure 9 diagrammatically shows the model of CS-C and the CS-C-based immunomodulatory process in wound healing.

## Conclusion

This study introduces a promising new biomaterial (CS-C), which is a TCM curcumin-regulated BMSC sheet. Curcumin improved the characteristics of the BMSC sheet, including BMSC activity, behavior, and ECM components. When it is applied for full-thickness cutaneous wound healing, CS-C possesses properties of immunoplasticity, which plays a vital role in immunoregulation during wound repair and promotes tissue reconstruction. In addition, the CS-C group had better engraftment since curcumin can enhance the delivery and efficacy of BMSCs to reinforce its pro-healing functions. These results demonstrate that the specific BMSC sheet induced with curcumin could be a promising biomaterial for adult full-thickness cutaneous wound healing, providing a new perspective for the future treatment of skin wound repair.

## Abbreviations

BMSC: Bone marrow-derived mesenchymal stem cell
CS-C: BMSC sheets induced by curcumin
CS-N: BMSC sheets induced without curcumin
ECM: Extracellular matrix
FN: Fibronectin
GFP: Green fluorescent protein
H&E: Hematoxylin and eosin
IFN: Interferon
IGF: Insulin-like growth factor
IL: Interleukin
IL1RN: Interleukin-1 receptor antagonist
PBS: Phosphate-buffered saline
PDGF: Platelet-derived growth factor
RT-PCR: Real-time polymerase chain reaction
SDF1: Stromal cell-derived factor 1
SEM: Scanning electron microscopy
SHG: Second-harmonic generation
TCM: Traditional Chinese Medicine
TGF: Transforming growth factor
TNF: Tumor necrosis factor
TPF: Two-photon fluorescence
VEGF: Vascular endothelial growth factor
